# Correlation study between increased fetal movement during the third trimester and neonatal outcome

**DOI:** 10.1186/s12884-019-2637-4

**Published:** 2019-12-04

**Authors:** Cuiqin Huang, Wei Han, Yajing Fan

**Affiliations:** Department of Obstetrics, Shanghai Jiao Tong University Affiliated Sixth People’s Hospital, 600# Yishan Road, Xuhui District, Shanghai, 200233 China

**Keywords:** Fetal movements, Neonatal outcome, Large for gestational age, Correlational study

## Abstract

**Background:**

We aimed to analyze the correlation between increased fetal movements in the third trimester and neonatal outcomes.

**Methods:**

We enrolled pregnant women (*n* = 219) who reported increased/excessive fetal movements in the third trimester in our hospital. A control group of healthy women (*n* = 278) who had undergone regular childbirth and delivery in our hospital during the same period and did not report abnormal fetal movements were also recruited. All pregnant women underwent fetal non-stress test. We analyzed the neonatal weight, appearance, pulse, grimace, activity, and respiration score, degrees of amniotic fluid contamination, amniotic fluid volume, conditions of umbilical cord around the neck and cord length, and incidence of small for gestational age. In addition, the incidence of preterm delivery, cesarean section rate, postpartum hemorrhage, and other postpartum complications were also analyzed. We then analyzed the correlation between increased/excessive fetal activity and neonatal outcomes.

**Results:**

Women with complaints of increased/excessive fetal movements exhibited increased fetal movements mainly around 31 and 39 weeks of gestation. Several pregnancy variables, including number of previous delivery, gestational age (less than 34 weeks and more than 37 weeks) and vaginal birth rate, were associated with increased/excessive fetal movements. In addition, women who reported increased/excessive fetal movements had higher odds of large for gestational age (LGA), particularly those with gestational age over 37 weeks.

**Conclusion:**

Increased/excessive fetal movements may be used to predict adverse neonatal outcome such as LGA.

## Background

Globally, 2.6 million infants were stillborn each year [1]. Hence, many efforts have been invested to study modifiable risk factors, which could be used as targets to introduce intervention to alleviate the risk of stillbirths. These efforts were mainly retrospective studies with case-controls to investigate risk factors such as fetal movements due to mother’s experience, sleep position during maternity, maternal intuition, exercise, and diet [2–4]. These studies have confirmed that fetal abnormalities can be detected early by monitoring fetal movement, which are a common complaint by pregnant women during the third trimester. Furthermore, these studies reported that reduced fetal movement is a clear maternal symptom associated with stillbirth and poor prognosis of neonates [5–8], possibly through placental dysfunction [9].

While reduced fetal movements often cause concern and anxiety among women and also required clinical assessments [6], clinical data regarding the relationship of increased/excessive fetal movements and prognosis of neonatal outcomes are currently not adequately available. A questionnaire study in Nigeria found that women tend to express concern on excessive movement in a significantly higher rate than on reduced movements (31.1% vs. 21.8%) [10]. Hence, it is important to study whether excessive fetal movements are associated with any adverse neonatal outcomes. This information would be used to guide medical professionals in providing nursing to women, and would also allow more detailed information to be provided to women in order to reduce maternal anxiety.

To this end, we analyzed the neonatal weight, appearance, pulse, grimace, activity, and respiration (APGAR) score, degrees of amniotic fluid contamination, amniotic fluid volume, conditions of umbilical cord around the neck and cord length, and incidence of small for gestational age of pregnant women, with or without complaints of increased fetal movements. In addition, the incidence of preterm delivery, cesarean section rate, postpartum hemorrhage, and other postpartum complications were also analyzed. We then reported the correlation between increased/excessive fetal activity and neonatal outcomes in this study.

## Methods

### Study participants

A cohort study was conducted by prospectively recruiting women who were admitted to the Emergency Department of Obstetrics, Shanghai Sixth People’s Hospital, Shanghai Jiao Tong University with a complaint of increased/excessive fetal movements from April 1st 2017 to November 30th 2017. Our Emergency Department provides 24-h service and has the capability to assess approximately 3000 women per year. We enrolled pregnant women (*n* = 219) who reported having increased/excessive fetal movements in the third trimester in our hospital. We also included a control group of healthy women (*n* = 278) who had undergone regular childbirth and delivery in our hospital during the same period and did not report abnormal fetal movements. All pregnant women underwent fetal non-stress test (NST).

Inclusion criteria were: 1) women over 28 weeks’ gestation; 2) with delivery record in our hospital during the study period. Exclusion criteria were: 1) women who had antenatally-diagnosed congenital anomalies and/or had multiple pregnancy; 2) with medical conditions requiring medication that could affect fetal movements during the study.

Fetal status was measure by attending staff using NST at our Obstetrics Emergency Department. After delivery, chart review was performed by the authors, and the pregnancy characteristics and outcomes were evaluated. Demographics data (e.g. marital status and maternal age) and characteristics related to pregnancy (e.g. number of previous deliveries, gestational age, maternal weight at last week of gestation) were collected, and several risk factors for fetal prognosis outcomes (e.g. hypertension more than 140/90 mmHg, and diabetes) were assessed. Finally, delivery (e.g. cesarean section rate, neonatal weight, APGAR score, degrees of amniotic fluid contamination, amniotic fluid volume, conditions of umbilical cord around the neck and cord length, and incidence of small for gestational age) and postpartum complications (e.g. postpartum hemorrhage, Neonatal Intensive Care Unit admission) information were also obtained. The APGAR score was determined by evaluating the newborn baby on five simple criteria on a scale from zero to two, then summing up the five values. The degrees of stained amniotic fluid were defined as follows:

1. Grade one: a small amount of meconium in a large amount of amniotic fluid, with slightly greenish or yellowish discoloration.
2. Grade two: a moderate amount of meconium in a fair amount of amniotic fluid, with clear meconium staining shown as ‘khaki green’ or brownish in color.
3. Grade three: a large amount of meconium in a reduced amount of amniotic fluid, with heavy meconium staining shown as thick with ‘pea soup’ consistency.

### Data analysis

Women with increased/excessive fetal movements were compared to the control group of women. Predictive Analytics Software 18 (IBM, 2009) was used for data analysis. Continuous data were analyzed using the *t*-test. Categorical data were analyzed using Pearson’s Chi-squared test. Multivariable logistic regression was then used to model the association between increased/excessive fetal movement and neonatal weight and pre-term delivery. In the model, we controlled for variables and covariates known to be associated with neonatal weight and pre-term delivery, including gestational age, maternal weight at last week of gestation, delivery mode, and hypertension.

## Results

**Fig. 1:**
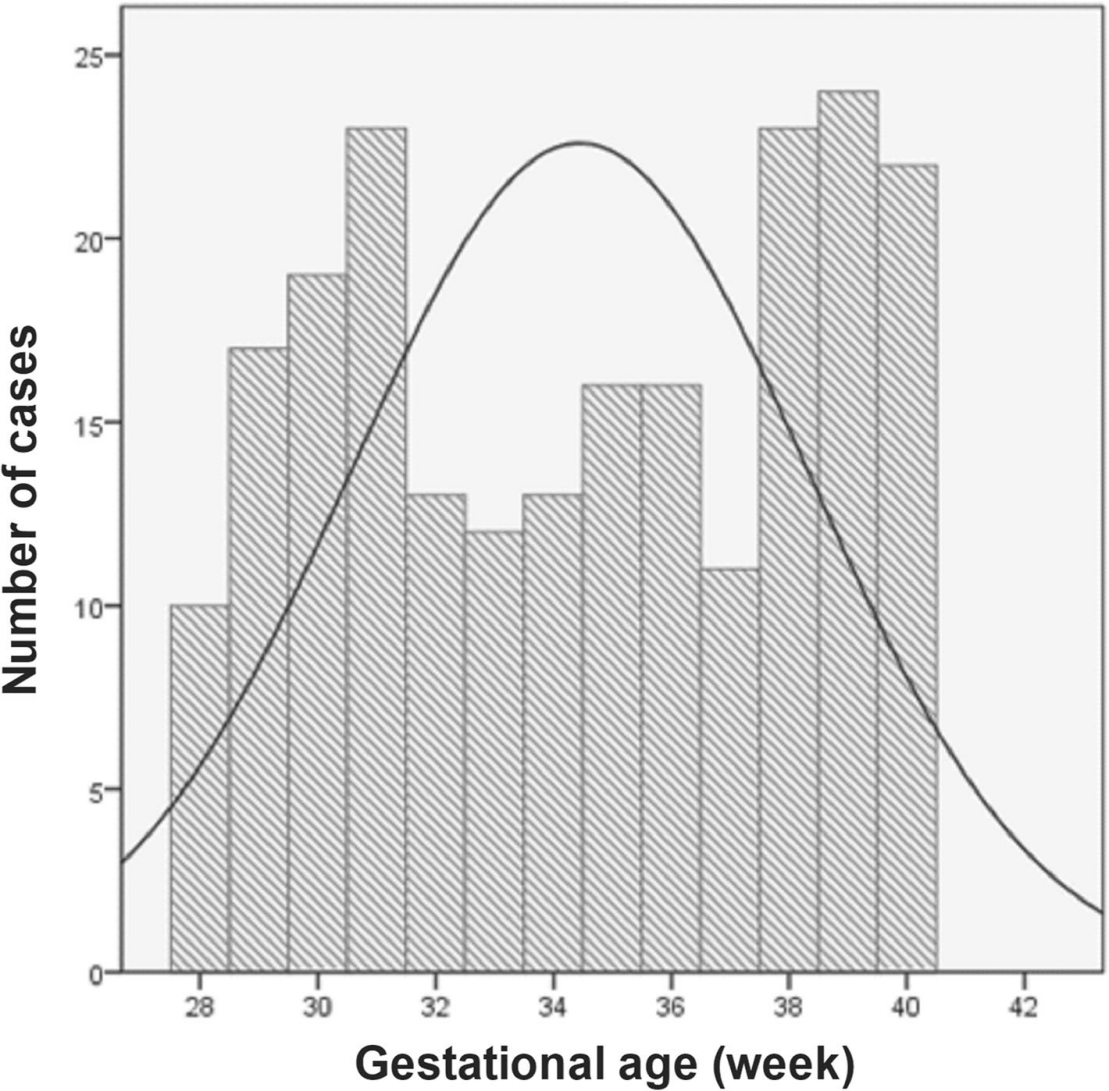
Number of women who experienced increased/excessive fetal movements during the third trimester of pregnancy

From April 1st 2017 to November 30th 2017, there were a total of 219 women recruited via the Obstetrics Emergency Department in our hospital with a complaint of increased/excessive fetal movements. We also included a total of 278 healthy women who had undergone regular childbirth and delivery in the Department of Obstetrics at our hospital during the same period and did not report abnormal fetal movements. As a result, we analyzed a total of 497 women who had delivery in our hospital. In general, about 9.6% of pregnant women who were treated in our hospital reported experience of increased/excessive fetal movements. The characteristics of the analytic cohort are shown in Table 1. Subjects were largely nulliparous (67.8%). We also found that hypertensive disease of pregnancy (12%) and diabetes (8%) were the most common comorbid conditions. The rates of pre-term, small-for-gestational-age neonates, postpartum hemorrhage (more than 500 ml), Neonatal Intensive Care Unit admission were relatively low. In women with a complaint of increased/excessive fetal movements, increased fetal movements appeared mainly between 31 and 39 weeks of gestation (Fig. 1).

**Table 1:** Characteristics of enrolled pregnant women

	Increased/Excessive Fetal Movements (*n* = 219)	Control (*n* = 278)	*P*
Age	30.8 ± 3.4	31.5 ± 5.1	0.061
Diabetes (Type I, II or GDM)	10 (4.5)	11 (3.8)	0.322
Hypertension: PIH, Chronic & Preeclampsia	16 (7.2)	22 (7.9)	0.412
Previous pregnancy			
0 (%)	192 (87.7)	145 (52.2)	0.001
1 (%)	27 (12.3)	126 (45.3)	
≥ 2 (%)	0 (0)	7 (2.5)	
Gestational age			
<34 week	2(0.9)	8(2.9)	0.002
34–37 week	5(2.3)	24(8.6)	
≥ 37 week	212(96.8)	246(88.5)	
Delivery mode			
Vaginal	140(63.9)	152(54.7)	0.043
C-section	79(36.1)	126(45.3)	
Postpartum hemorrhage volume (mL)	317.5 ± 324.8	349.2 ± 266.4	0.233

*GDM* Gestational Diabetes Mellitus, *PIH* pregnancy-induced hypertension

Using chi-square tests, we found that several pregnancy variables were associated with increased/excessive fetal movements (Table 2): number of previous delivery [odds ratio (OR) 2.2, 95% confidence interval (CI) 1.5–3.0], gestational age (less than 34 weeks and more than 37 weeks; OR 2.1, 95% CI 1.4–3.1) and vaginal birth (OR 1.7, 95% CI 1.1–2.9). In addition, we found that women who experienced increased/excessive fetal movements had less incidence of pre-term delivery (OR 1.4, 95% CI 1.0–2.1), and the newborns had higher neonatal weight (OR 2.5, 95% CI 1.6–3.2) (Table 2).

**Table 2:** Characteristics of neonates

	Increased/Excessive Fetal Movements (*n* = 219)	Control (*n* = 278)	*P*
Pre-term neonates	7 (3.2)	32 (11.5)	0.001
APGAR score			
8–10	218 (99.5)	274 (98.6)	0.205
5–7	0 (0)	3 (1.1)	
0–4	1 (0.5)	1 (0.4)	
Umbilical cord around the neck			
0	151 (68.9)	185 (67.3)	0.160
1	59 (26.9)	68 (24.5)	
≥ 2	9 (4.1)	23 (8.3)	
Amniotic fluid contamination			
No	201 (91.8)	247 (88.8)	0.454
I degree	10 (4.6)	21 (7.6)	
II degree	4 (1.8)	3 (1.1)	
III degree	4 (1.8)	7 (2.5)	
NICU admission	1 (0.4)	1 (0.3)	0.655
Neonate weight (g)	3450.7 ± 425.6	3286.1 ± 546.4	0.001
Neonate weight			
SGA	18 (8.4)	25 (9.1)	0.232
LGA	22 (10.3)	17 (6.3)	0.012
AGA	179 (81.3)	236 (84.6)	0.124

*SGA* Small for Gestational Age, *AGA* Average for Gestational Age, *LGA* Large for Gestational Age

In the multivariable model estimating the association between increased/excessive fetal movement and neonatal outcomes (Table 3), there were higher odds of large for gestational age (LGA) in women who had increased/excessive fetal movements (adjusted OR 1.86, 95% CI 1.69–2.07). Higher odds of LGA were also associated with women who had increased/excessive fetal movements and gestational age more than 37 weeks (adjusted OR 1.98, 95% CI 1.35–2.24).

**Table 3:** Logistic regressions modeling effect of fetal movements and gestational age on neonatal outcome [adjusted odds ratios (95% confidence intervals)]

	LGA *N* = 219
Duration for 10 Fetal Movements	
≥ 30 min	Reference
10–30 min	1.09 (0.89–1.34)
< 10 min	1.86 (1.49–2.07)*
Gestational age	
< 34 weeks	1.01 (0.81–1.82)
34–37 weeks	Reference
≥ 37 weeks	1.98 (1.35–2.24)*

Models are adjusted for age group, gestational weight gain, delivery history and pre-pregnancy diabetes or hypertension. ^*^p < 0.05

## Discussion

In the present study, we found that the percentage of women who reported increased/excessive fetal movements was relatively higher in those who had the first-time pregnancy compared the control group. This incidence rates dropped in those who had a history of pregnancy. This phenomenon may be due to the first pregnancy, during which pregnant women are not experienced in the method of counting fetal movement and appear more sensitive and cautious during counting. Hence, these first-time pregnant women may report increased fetal movements more frequently, which might compromise the objectivity of data in the current study. In fact, increased/excessive fetal movement is quite a common experience after 37 weeks of gestation. Therefore future investigations should include more objective measurements to address this limitation.

One important finding in our study is that, most of the neonates with increased fetal movement did not show poor prognosis. Instead, we observed better prognosis, which was reflected in the lower percentage of pre-term infants in women who had increased fetal movement. The neonatal weight of the newborns was significantly higher in women who had increased fetal movement than those of the control group. However, this may be also due to a higher percentage of pre-term infants in the control group, resulting in a decrease in the weight of newborns in the control group. Furthermore, in women who had increased fetal movements, we observed that increased fetal movements appeared mainly between 31 and 39 weeks of gestation. This increased fetal movement at 31 weeks of gestation is likely because that fetal weight gain is in an accelerated period, when the fetal movement may be frequent and obvious. At the 39th week of pregnancy, the frequency of false contractions increased, coupled with the proximity of childbirth. These factors may also contribute to the enhanced consciousness of fetal movement in women. Furthermore, our findings that about 9.6% pregnant women reported experience of increased/excessive fetal movements were similar with previous reports. In the situation, task, actions, and results (STARS) cohort study, 1714 women were recruited from more than seven countries, and 8.5% of respondents reported excessive fetal movements [11]. Among the respondents, the symptom frequency from the four major countries who participated in the survey remained consistent. Similarly, the frequency of perceived excessive fetal movements was close to that of the 10% of women who was analyzed in a stillbirth study in Sweden [12]. Interestingly, similar to our report, the perception of excessive fetal movements was reported more frequently (12% of respondents) when gestation was beyond 37 weeks [12]. Together with our reports, these results suggested that women in the third trimester of pregnancy may encounter more frequent excessive fetal movements.

Another important finding in our study is that, our regression analysis found that higher odds of LGA were associated with women who had increased/excessive fetal movements and gestational age over 37 weeks. One of the primary risk factors of LGA is poorly-controlled diabetes, particularly gestational diabetes, as well as pre-existing diabetes mellitus [13–16], which increase maternal plasma glucose and insulin levels, as well as stimulates fetal growth. In addition, studies have shown that gestational age more than 40 weeks and excessive maternal weight gain can increase incidence of LGA [15]. Consistent to this literature, our study confirmed that gestational age over 37 weeks was associated with increased incidence of LGA. Furthermore, women with increased/excessive fetal movements also had higher incidence of LGA, particularly when the gestational age was more than 37 weeks.

Changes in amniotic fluid may indicate fetal intrauterine hypoxia, fetal acidosis in the fetus, and the abnormal fetal growth [17, 18]. Surprisingly, we did not observe any difference in amniotic fluid volume or degrees of stained amniotic fluid in women who had increased fetal movement when compared to those of the control group. In addition, there was no correlation between increased fetal activity and the incidence of umbilical cord around the neck. These results suggested that amniotic fluid volume and contamination and the incidence of umbilical cord around the neck may not be the contributing factors in increased fetal movements.

While our study did not report increased cases of stillbirth in women who experienced increased/excessive fetal movements. Case-control studies have been performed in order to examine if there is any difference in the frequency of increased/excessive fetal movements between pregnancies with live births and those with stillbirth. In the Auckland Stillbirth Study, women who had stillbirth experienced more single episodes of excessive fetal activity [19]. However, more than one episode of vigorous fetal activity was less likely perceived by those who had stillbirth. In addition, women who had stillbirth were less likely to report the perception of increased fetal movements compared to controls [19]. Furthermore, one episode of vigorous activity was more likely perceived by women who had stillbirth in the STARS study [20]. Hence, these results suggest that a sudden episode of excessive fetal activity may indicate the compromise of fetus that is related to the disturbance of environment in uterus. Future studies with a larger cohort will be necessary to examine this hypothesis.

## Conclusion

Our study is the first prospective study to investigate the correlation between incidence of excessive fetal activity and neonatal outcomes. Historically, the uncertainty regarding measuring and reporting excessive fetal movements has led to focusing on reduced fetal movement and its effects on perinatal mortality and morbidity [21, 22]. While our study found no evidence for the association of excessive fetal movement with perinatal mortality, we did report adverse neonatal outcome (i.e., LGA) was correlated with increased/excessive fetal movements, particularly when the gestational age was more than 37 weeks. An improved understanding into the cause of excessive fetal activities and outcomes would allow future studies to investigate whether reduced adverse neonatal outcomes can be achieved by encouraging women to report excessive fetal activities followed by appropriate intervention. This would also provide translational value to modifiable risk factors for improving perinatal and neonatal outcomes.

## Data Availability

The datasets generated and/or analysed during the current study are not publicly available due to results are obtained from identifying images and/or other clinical details of participants that may compromise anonymity, but are available from the corresponding author on reasonable request.

## Abbreviations

APGAR: Appearance, pulse, grimace, activity, and respiration
CI: Confidence interval
LGA: Large for gestational age
NST: Non-stress test
OR: Odds ratio
STARS: Situation, task, actions, and results
